# Effectiveness of a brief community outreach tobacco cessation intervention in India: a cluster-randomised controlled trial (the BABEX Trial)

**DOI:** 10.1136/thoraxjnl-2016-208732

**Published:** 2016-10-05

**Authors:** Bidyut K Sarkar, Robert West, Monika Arora, Jasjit S Ahluwalia, K Srinath Reddy, Lion Shahab

**Affiliations:** 1 Public Health Foundation of India, New Delhi, India; 2 Department of Epidemiology and Public Health, Cancer Research UK Health Behaviour Research Centre, University College London, London, UK; 3 School of Public Health, Rutgers University, New Brunswick, USA

**Keywords:** Smoking cessation, Tobacco control

## Abstract

**Background:**

Tobacco use kills half a million people every month, most in low–middle income countries (LMICs). There is an urgent need to identify potentially low-cost, scalable tobacco cessation interventions for these countries.

**Objective:**

To evaluate a brief community outreach intervention delivered by health workers to promote tobacco cessation in India.

**Design:**

Cluster-randomised controlled trial.

**Setting:**

32 low-income administrative blocks in Delhi, half government authorised (‘resettlement colony’) and half unauthorised (‘J.J. cluster’) communities.

**Participants:**

1213 adult tobacco users.

**Interventions:**

Administrative blocks were computer randomised in a 1:1 ratio, to the intervention (16 clusters; n=611) or control treatment (16 clusters; n=602), delivered and assessed at individual level between 07/2012 and 11/2013. The intervention was single session quit advice (15 min) plus a single training session in yogic breathing exercises; the control condition comprised very brief quit advice (1 min) alone. Both were delivered via outreach, with contact made though household visits.

**Measurements:**

The primary outcome was 6-month sustained abstinence from all tobacco, assessed 7 months post intervention delivery, biochemically verified with salivary cotinine.

**Results:**

The smoking cessation rate was higher in the intervention group (2.6% (16/611)) than in the control group (0.5% (3/602)) (relative risk=5.32, 95% CI 1.43 to 19.74, p=0.013). There was no interaction with type of tobacco use (smoked vs smokeless). Results did not change materially in adjusted analyses, controlling for participant characteristics.

**Conclusions:**

A single session community outreach intervention can increase tobacco cessation in LMIC. The effect size, while small, could impact public health if scaled up with high coverage.

**Trial registration number:**

ISRCTCN23362894.

**Key messages**

**What is the key question?**

- Does a potentially low-cost and scalable community-outreach intervention, single session advice (15 min) to stop tobacco use combined with training in yogic breathing exercise, increase abstinence rates from tobacco use in the context of a low–middle income country (LMIC) compared with very brief quit advice (1 min) alone?

**What is the bottom line?**

- A simple, cheap and potentially scalable community-outreach intervention increases smoked and smokeless tobacco cessation in India, producing an effect size similar to that of brief physician advice in high-income countries.

**Why read on?**

- Given the number of tobacco users and limited resources in LMIC, there is a need to develop cheap and scalable interventions to combat tobacco use and, to our knowledge, this is the first trial to establish the effectiveness of a single session community outreach intervention to stop tobacco use in low-income communities in any LMIC worldwide.

## Introduction

Tobacco use causes six million premature deaths each year, most in low-income and middle-income countries (LMICs),^1^ and one million in India alone.^2^ Even small increases in long-term tobacco cessation rates can have an important public health impact.^3^ Combatting the tobacco epidemic requires a comprehensive, global strategy including taxation, smoke-free policies, and dedicated healthcare services to encourage and support cessation.^4^ However, implementation in LMICs has been limited, hampered by weak regulatory and enforcement infrastructure.^5^ Cheap, scalable tobacco cessation interventions that build on existing infrastructure could strengthen tobacco control in the Indian context.^6^

Brief opportunistic advice from a health worker has been found to be a highly cost-effective intervention to promote smoking cessation in high-income countries (HICs),^7^ increasing 12-month quit rates by one to three percentage points^8^ and offering assistance is an important part of the process.^9^ However, there is limited evidence on the effectiveness of quit advice in LMICs, from non-physicians, or for smokeless tobacco use. These represent important gaps in the evidence base. This paper attempts to fill these gaps, evaluating an intervention of single session smoked and smokeless tobacco cessation advice plus a single session of yogic breathing exercises, delivered by healthcare professionals via community outreach in India.

Tobacco use, a major cause of health inequalities, is more prevalent and quit success rates are lower in deprived groups.^10^ Even within poorer countries, there is a gradient in tobacco use.^11^ While there is a need to improve support across all societal strata in LMICs, arguably, it is particularly important to focus on low-income communities within those countries by outreach to involve tobacco users who would not otherwise come into contact with health services.^6^

Given limited resources, and a lack of affordable pharmacotherapy with proven effectiveness for smokeless tobacco users, providing assistance presents a challenge in LMICs such as India with more smokeless tobacco users than smokers.^12^ A promising and culturally appropriate option in this context is the use of yogic breathing exercises, known as ‘Pranayama’. These are easy to learn and practice, and there is preliminary evidence they can reduce cigarette cravings^13^ ^14^ and promote quitting.^15^ We therefore developed a brief community outreach intervention that included training on simple breathing exercises to control cravings to aid cessation.

Ideally, an evaluation of a multi-component intervention would involve multiple experimental conditions allowing determination of active components. Unfortunately, with limited resources the main priority is to find an intervention that works. Understanding how it can be optimised can be done once this ‘base camp’ has been established. Therefore we opted for a pragmatic, effectiveness trial of an intervention compared with the closest to ‘usual care’ that was ethically acceptable, undertaken in conditions mimicking what is feasible in routine practice. Cluster rather than individual randomisation was chosen to minimise risk of contamination given the close-knit nature of these communities. Both treatment delivery and assessment were at an individual level. Following guidelines on complex intervention development and evaluation,^16^ this study built on the extensive work on brief advice from HIC, and proof of concept studies on breathing exercises as well as field studies in this particular setting.^17^ Specifically, the present study sought to answer the following research question: how effective is single session quit advice (15 min) with instruction on simple breathing exercises compared with very brief advice (VBA) alone (1 min) in promoting tobacco cessation when delivered by outreach workers in low-income communities in India?

## Methods

### Study design

This pragmatic, cluster-randomised controlled trial set in low-income communities (urban slums) compared two treatments: the intervention arm included a single face-to-face session of quit advice plus a single training session on yogic breathing exercises (BA-YBE); the control arm comprised VBA alone. Assessments were performed at baseline (immediately before treatment delivery), 4 weeks and 7 months after delivery of the treatments. The study was approved by the UCL (3051/002) and Public Health Foundation of India (TRC-IEC-122/11) ethics committees.

### Participants

Eligible participants were any current, daily, adult tobacco user aged 23 years or above, living in selected low-income communities, who provided consent to participate. Recruitment into the study started in July 2012 and the trial concluded in November 2013. The proposed study area contained approximately 36 000 adults living in 32 randomly selected administrative blocks of large communities spread over a wide geographical area in Delhi, India. These low-income communities had been selected for a previous tobacco study conducted among youths aged 10–19 years in 2009,^18^ therefore only adults aged 23 or above were eligible to avoid contamination with the previous study.

### Intervention

Participants in the intervention condition received a single session of tobacco quit advice lasting an average of 15 min, and short training in two yogic breathing exercises. The quit advice contained behaviour change techniques which have been shown to improve quit rates,^19^ including coping training, medication advice, social support and relapse prevention. Two breathing exercises which are easy to learn and practise, ‘Kapalbhati’ (normal deep inhalation-forceful exhalation) and ‘Anulom vilom’ (alternate nostril breathing), were chosen as they are culturally appropriate and have been shown to control cravings.^14^ A written standard operating procedure was followed by the research team, including a script for quit advice and a standard video for training on yogic breathing exercises to increase fidelity. Further details are available in the published protocol.^20^

### Control

Whilst a control condition with no advice on tobacco use (‘usual care’) would be preferable to assess the full intervention effect, this was considered unethical. Therefore a single control session involving very brief quit advice^21^ was used. This provided verbal information about the harmful effects of tobacco use and advice to stop tobacco use and lasted on average 1 min.

### Procedure, randomisation and masking

A census survey of adults in urban low-income communities of Delhi was conducted to establish an appropriate sampling frame for this study.^17^ As low-income blocks in the study area are stratified into two community types (government authorised ‘resettlement colonies’ and unauthorised settlements called ‘Jhuggi-Jhopri’ or ‘JJ clusters’), an equal number of both were identified for inclusion. The clusters were identified and a list of all eligible tobacco users finalised within each cluster prior to randomisation of clusters to the intervention or control arm. The random sequence was computer generated and blocked to ensure equal numbers of each community type in the intervention and control conditions. Based on the random sequence, 16 clusters (8 from each community type) were allocated to the treatment (BA-YBE) and control (VBA) arm, respectively. While participants were blind to allocation, being a cluster-randomised trial, concealment of allocation to the research team was not feasible.

The baseline interview was conducted at the doorstep of potential participants and those identified as tobacco users were invited to participate. Each cluster was divided into three to four geographical parts (streets/lanes/outer/inner) comprising an equal number of households from which an equal number of participants were approached. Those willing to provide consent were checked for eligibility and formal written consent was obtained. In the case of multiple eligible participants in the same household, the first consenting tobacco user identified in the sampling frame was recruited.

The treatment delivery team consisted of two members, a medical graduate researcher (physician) and a field investigator (community health worker) trained and initially supervised by the medical graduate researcher. The intervention was delivered by either of them. The field investigators were given 1 day f classroom training for intervention delivery using standard tools like a script for quit advice and use of a standard video for training in yogic breathing exercises. All content was delivered face to face in non-technical, local language (Hindi) in both groups. Follow-up visits comprised face-to-face interviews at the residence of participants, 4 weeks and 7 months after the baseline visit. Due to cluster randomization, blinding was not feasible but follow-up was carried out by a different team of field investigators.

### Measures

All measures, including outcome measures, were pre-specified and defined in the ISRCTN Registry and published protocol.^20^ The primary outcome measure, 6-month sustained abstinence, was defined as per Russell Standard (RS) criteria,^22^ allowing a maximum of five instances of tobacco use (in whichever form) in the 6 months preceding the follow-up conducted 7 months after treatment delivery, and was biochemically validated. As the sample included smokeless and smoked tobacco users, salivary cotinine was used to detect tobacco exposure, assessed by ELISA with a cut-off of 20 ng/mL, equivalent to the 13 ng/mL used for the gas chromatography mass spectrometry method.^23^ Self-reported abstainers above the cut-off were considered to be continuing tobacco users as were participants lost to follow-up, in accordance with the intention-to-treat principle and RS criteria.^22^ Nicotine replacement therapy (NRT) and other medication use was assessed at follow-up to exclude false-positive cotinine results.

The secondary outcome measure was 7-day biochemically validated point prevalence abstinence assessed 7 months after treatment delivery. At the 4-week follow-up, all participants were asked whether they had used tobacco in the past 7 days (7-day point prevalence), and intervention group participants were also asked how often they had used the yogic breathing exercises and how useful they had found them on a seven-point scale, ranging from ‘not at all’ to ‘very helpful’.

At baseline, standard socio-demographic and smoking characteristics were assessed. In addition to age, sex and marital status, participants were asked about employment status (employed vs other), highest educational attainment (at least primary education vs not), caste (lower caste vs other) and household income (≤5000 vs >5000 rupees/month). Participants were asked when they started tobacco use, dependence (assessed with the heaviness of smoking index,^24^ modified for smokeless tobacco users), quit attempts in the past year, length of the attempt and whether support was used as well as how confident they felt about stopping smoking on a seven-point scale ranging from ‘not at all confident’ to ‘very confident’. Lastly, adverse and serious adverse events were monitored and recorded by the medical graduate researcher throughout the study.

### Sample size

The initial power calculation based on 32 clusters indicated that 31 participants per cluster would be needed for 90% power to detect a 5% difference in quit rates between the treatment and control group in two-tailed analysis (7% vs 2%).^20^ The effect estimate was based on early, similar work^25^ and assumed an intra-cluster correlation co-efficient (ICC) of 0.01, typical in this kind of study, and a design effect of 1.24 to account for cluster randomisation. However, as low-intensity interventions can have substantial attrition rates (in trials using evidence-based methods to increase response rates, dropout rates of 20–25% at 6-month follow-up are common)^26^ which reduces the power to detect effects in an intention-to-treat design^27^ and due to the uncertainty around the ICC estimate, we over-recruited by 20% per cluster (37.2 participants, rounded up to 38 participants), resulting in a final target sample size of 1216 (38×32 clusters). This sample size also provided 90% power to detect a larger difference (8%) in subgroup analyses.

### Analysis

Primary and secondary smoking cessation outcomes and baseline group differences were analysed with a mixed-effects log-binomial or linear regression with appropriate link functions for categorical (log/logit-binomial) and continuous (identity Gaussian) outcome variables using the ‘lme4’ package in R (V.3.2.0) (Bates DM. lme4: Mixed-effects modelling with R: Springer, 2010) to account for clustered observations, which is preferable to the protocol-specified complex samples analysis.^28^ All other analyses were conducted in STATA (V.13.1). Results from the conditional/cluster-specific models were confirmed with marginal/population-averaged models using generalised estimating equations (‘xtgee’ command) with an exchangeable correlation matrix to account for clustering,^29^ and ICC was determined using the analysis of variance method.^30^

In planned subgroup analyses, intervention by participant (smoked vs smokeless tobacco and dual product users) and intervention by provider (trained health professional vs trained field health workers) interaction terms were included in models to assess homogeneity of the effect across these subgroups. In sensitivity analysis, we analysed only those who actually received the treatment and effect estimates were adjusted for covariates to account for chance imbalances in baseline characteristics between intervention and control conditions. Significance levels for multiple comparisons were adjusted with the Benjamini-Hochberg procedure.^31^ The study was registered with the International Standard Randomised Controlled Trial Number Registry (number ISRCTCN23362894).

## Results

**Figure 1:**
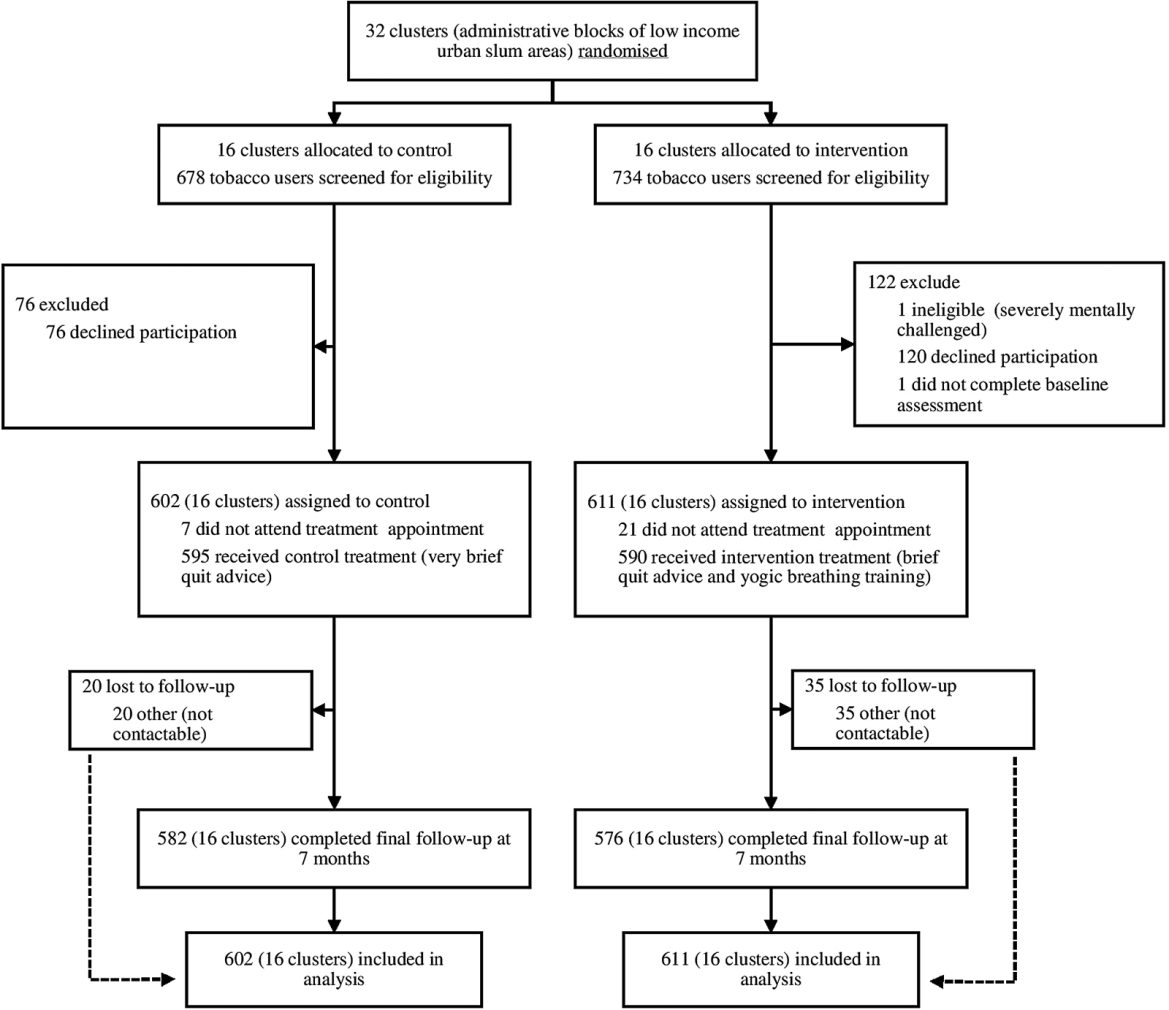
Numbers of participants and clusters enrolled in the study and included in the primary analysis.

As shown in figure 1, 85.9% (1213/1412) of eligible tobacco users approached between January 2012 and November 2013 agreed to take part in the study; the most common reason for not taking part was lack of interest. The follow-up rate at final endpoint was high at 95.3% (1158/1213) and did not differ between arms. There were no differences in baseline characteristics between those who were and were not followed up.

The sample of tobacco users recruited was largely male, middle aged, married, in employment, with at least a primary education, most were relatively deprived as measured by household income and nearly half were from the lower caste (table 1). There were slightly more tobacco smokers than smokeless tobacco users; a substantial proportion were dual users. Most had been using tobacco products for 20 years and were moderately dependent. A fifth had made a quit attempt in the last year, and past quit attempts had lasted up to 2 months, but very few had previously used any quit support. Participants were generally confident in their ability to quit. There were no chance imbalances between groups with the exception of caste: significantly more participants in the intervention than control group were lower caste (table 1).

**Table 1:** Baseline characteristics of participants

Characteristics	Total (N=1213)	Intervention (BA-YBA)* (N=611)	Control (VBA)† (N=602)
Sociodemographic			
% (N) Male	79.7 (966)	77.4 (472)	82.1 (494)
Mean (SD) age in years	46.3 (13.6)	45.2 (12.8)	47.4 (14.2)
% (N) Married/cohabiting‡	86.4 (1044)	85.2 (517)	87.5 (527)
% (N) In employment§	70.7 (856)	71.7 (438)	69.7 (418)
% (N) No primary education¶	35.3 (426)	33.3 (202)	37.3 (224)
% (N) Household income ≥5000 INR/month**	34.1 (403)	35.7 (212)	32.5 (191)
% (N) Lower caste††	43.8 (512)	50.7 (290)	37.2 (222)
Tobacco use			
% (N) Smokers	63.8 (774)	65.0 (397)	62.6 (377)
% (N) Smokeless users	58.5 (710)	58.8 (359)	58.3 (351)
% (N) Dual users	22.3 (271)	23.7 (145)	20.9 (126)
Mean (SD) age started use‡‡	22.4 (9.6)	22.2 (10.0)	22.6 (9.3)
% (N) Made quit attempt in the previous year§§	20.0 (229)	22.3 (126)	17.8 (103)
Mean (SD) length of longest quit attempt (in weeks)¶¶	7.6 (30.0)	7.5 (33.2)	7.8 (26.5)
% (N) Previously used support in a quit attempt***	3.8 (46)	3.8 (23)	3.8 (23)
Mean (SD) heaviness of smoking index score (0-6)†††	3.0 (1.7)	3.2 (1.6)	2.8 (1.7)
% (N) Usually uses tobacco within 5 min of waking‡‡‡	38.0 (458)	40.0 (243)	36.0 (215)
Mean (SD) confidence in stopping score (1–7)§§§	4.4 (1.9)	4.5 (1.9)	4.2 (2.0)

Different pairs of superscript letters indicate a significant group difference (p<0.05) after adjustment for false discovery rate and accounting for clustered nature of data.

*Brief quit advice and yogic breathing exercises.

†Very brief quit advice.

‡Four cases missing/refused to answer.

§Three cases missing/refused to answer.

¶Five cases missing/refused to answer.

**Thirty-two cases missing/refused to answer.

††Forty-five cases missing/refused to answer.

‡‡Twenty-nine cases missing/refused to answer.

§§Sixty-nine cases missing/refused to answer.

¶¶Twenty-six cases missing/refused to answer.

***Seventeen cases missing/refused to answer.

†††Sixteen cases missing/refused to answer.

‡‡‡Nine cases missing/refused to answer.

§§§Eight cases missing/refused to answer (scale from 1 ‘not at all confident’ to 7 ‘very confident’).

Regarding the primary outcome, 2.6% (16/611) of participants were continuously abstinent at 6 months as per RS criteria, validated by saliva cotinine. Intention-to-treat analysis showed that participants in the intervention group were about five times more likely to be abstinent at 6 months than those in the control group; absolute abstinence rates were increased by 2% (table 2), yielding a number needed to treat of 48 to produce an additional quitter. This difference did not materially alter and remained significant when controlling for all other covariates.

**Table 2:** Effect of intervention on biochemically verified smoking cessation*

	Intervention (BA-YBE)	Control (VBA)			
	% (number)		Percentage point difference (95% CI)	Intracluster correlation coefficient (95% CI)	Model 1: Relative risk† Model 2: Adj. relative risk‡ Model 3: Adj. relative risk§ (95% CI)
Primary outcome: abstinence for 6 months¶	2.6 (16/611)	0.5 (3/602)	2.12 (0.74 to 3.51)	0.014 (0.000 to 0.033)	Model 1: 5.32 (1.43 to 19.74) ** Model 2: 5.10 (1.46 to 17.84) ** Model 3: 4.54 (1.21 to 17.01) ††
Secondary outcome: point prevalence at 6 months‡‡	3.1 (19/611)	0.8 (5/602)	2.28 (0.72 to 3.83)	0.014 (0.000 to 0.034)	Model 1: 3.77 (1.31 to 10.81) ** Model 2: 3.71 (1.25 to 11.02) §§ Model 3: 2.87 (0.92 to 8.93) ¶¶

*Outcome measures were biochemically verified using saliva cotinine; all participants reporting no tobacco use at final follow-up were asked to provide a saliva samples. Failure to provide biochemical verification was low (1.2% (14/1213); there were no differences by treatment group) with those not providing saliva samples or lost to follow-up counted as treatment failures.

†Results from conditional/cluster-specific approach with mixed effects log-binomial regression are presented, using intention to treat analysis based on all participants (N=1213). Results were confirmed using a marginal/population approach which yielded comparable estimates (primary outcome: 5.33, 95% CI 1.30 to 21.90, p=0.020; secondary outcome: 3.76, 95% CI 1.31 to 10.82, p=0.014).

‡Results from model 1 were adjusted for chance imbalances, including all variables in table 1. Data missing at random were imputed using standard multivariate imputations with chained equations (MICE) in STATA (‘mi chained’ command with burn in of 100 iterations and 20 imputations) which is more suitable for mixed (dichotomous and continuous) variables,^32^ and this analysis is therefore based on all participants (N=1213).

§Results from model 1 were adjusted for chance imbalances, including all variables in table 1. Only participants with complete data were included in the analysis (N=1009).

¶The primary outcome conforms to the Russell Standard guidelines, that is, self-reported abstinence for at least 6 months (with no more than five occasions of tobacco use) and no tobacco use in the last week, as indicated at final follow-up.

**p=0.01.

††p=0.03.

‡‡The secondary outcome was point prevalence at 6 months defined as complete abstinence in the week prior to follow-up.

§§p=0.02.

¶¶p=0.07.

BA-YBE, brief advice, yogic breathing exercises; VBA, very brief advice.

Validated 7-day point prevalence at 6 months in the total sample was marginally higher at 2.0% (24/1213). Participants in the intervention group were nearly four times more likely to achieve 7-day abstinence at 6 months than those in the intervention group and as before results did not materially change when controlling for other covariates (table 2).

Overall self-reported 7-day point prevalence at 4 weeks was 9.3% (113/1213). As with the primary and secondary outcomes, abstinence rates were significantly higher in the intervention group than the control group, more than doubling (RR 2.03; 95% CI 1.30 to 3.19; p=0.002) to 12.4% (76/611) from 6.1% (37/602). At 4-week follow-up, 61.1% (373/611) in the intervention group had continued to use the breathing exercises, a fifth (19.0% (116/611)) on most days. They rated the breathing exercises as helpful (mean=5.3, SD=1.8). No adverse or serious adverse events were reported.

In pre-specified per protocol analyses of the primary outcome, we analysed data from participants who had actually received treatment, given that significantly more participants in the intervention group than the control group (3.4% (21/611) vs 1.2% (7/602); p=0.03) missed their treatment. This did not alter the effect of the intervention (RR 5.48; 95% CI 1.46 to 20.51; p=0.01). We also included potential effect modifiers, the type of tobacco used (smoked vs smokeless, excluding dual users), poly-use (dual vs single use) and intervention provider (trained health professional vs trained field health workers), as interaction terms in the main model to assess whether effects differed between these groups. There was no evidence that type of product used (p_interaction_=0.32), poly-use (p_interaction_=0.85) or the intervention provider (p_interaction_=0.90) affected the outcome.

## Discussion

In a trial of tobacco users from low-income communities in India, 6-month sustained biochemically verified abstinence rates were increased fivefold by a low-cost intervention combining single session quit advice with yogic breathing exercises when compared with very brief quit advice alone. The increase of 2% in absolute quit rates is close to that obtained by other low-intensity interventions observed in HIC.^8^ ^33^ ^34^ These results also compare favourably with previous evaluations of more intensive interventions in LMIC which roughly double short-term abstinence rates from tobacco.^5^ While the absolute quit rates were lower than expected, this may not only reflect the simplicity of the intervention which did not provide any pharmacological support but also the broader challenge of making behaviour change interventions relevant in the context of extreme deprivation, as has been observed elsewhere.^35^ However, since it is non-physician based, it is potentially scalable in settings like India with an inadequate healthcare delivery system especially for populations with limited access to physicians or medications. It is noteworthy that the average family income of participants was £50 per month or less than £2 per day, which makes even NRT unaffordable unless funded by the government. At follow-up, use of NRT and other medications for quitting was assessed but not reported by any participants, likely due to prohibitive costs.^36^

To our knowledge, this is the first trial using a rigorous design, including longer-term, biochemically validated outcomes to establish the effectiveness of brief, pro-active quit advice in low-income communities in any LMIC. Given that community health workers, who earn around £100–150 per month, were given just 1 day training to deliver the intervention, that the intervention was equally effective when provided either by a community health worker or by a physician researcher and that total study material costs were below £200, this intervention is likely to also be highly cost effective at a cost of less than £10 per treated tobacco user.^37^

This study benefited from assessing an evidence-based and theory-based intervention using standardised methodology, including validated longer-term outcomes, extensive prior field work and low attrition rates. A number of factors may have contributed to the effectiveness and reach of the intervention. First, yogic breathing exercises in addition to reducing cravings,^14^ possibly via actions in the insula in the cerebral cortex,^38^ may also appeal due to being a novel, culturally acceptable cessation aid which provides an element of support without the need for referral or expensive medications. Second, the proactive, convenient delivery of the intervention near participants' homes may have engaged more tobacco users. Third, the treatment being free of charge may have drawn in tobacco users, irrespective of their readiness to quit. Lastly, the advice, being centred around evidence-based content,^19^ was tailored to the specific cultural context, targeting users of both smoked and smokeless tobacco, and—as evidenced by very limited smoking-related knowledge at baseline (not reported here)—provided extensive details hitherto unknown to participants.

There were some limitations. Since the intervention comprised both quit advice and training in yogic breathing exercises, the effect of specific intervention components cannot be dissociated. However, the priority at this stage was to identify a potentially cost-effective, scalable brief community outreach intervention that included effective, research-based components. Future factorial studies should further isolate the impact of different intervention components on outcome. The number of events in this trial was small. Although this is not uncommon in low-intensity interventions, replication of findings is warranted. Lastly, whilst every effort was made to select a random sample, findings are necessarily limited to this sample of deprived tobacco users in Delhi. However, given that outcomes were comparable to those from a study evaluating a similarly brief, two-session tobacco use intervention in rural India,^39^ the results may be generalised to other geographic locations and LMICs. Nonetheless, further work should confirm whether this type of opportunistic, brief quit advice and yogic breathing exercises is equally effective in other relevant settings, such as schools or work places, to maximise its potential impact.

In conclusion, we have shown a substantial effect of a potentially inexpensive, scalable, non-physician-dependent, culturally sensitive intervention to aid cessation of tobacco use in LMICs. Although the number needed to treat to gain an additional quitter was high at 48, the low intervention cost and ease of implementation with limited additional resources (see protocol for details)^20^ means that it may offer a credible alternative to more effective but also more expensive and intensive treatments, such as pharmacotherapy, with very low coverage, to tackle the enormous tobacco problem facing disadvantaged users in some of the poorest countries of the world. Despite the small effect observed, even minimal changes in tobacco use can have clinical significance.^3^ Indeed, if this intervention were to be rolled out across India, it would likely result in several million fewer tobacco users per year. While further research is needed to confirm our findings in different settings, the current trial is consistent with results from HIC and underlines the great potential of low-intensity interventions to improve public health in the specific context of LMICs.
